# Supplementary material for: Resolving the full spectrum of human genome variation using Linked-Reads
Source: Genome Res. 2019 Apr;29(4):635–45. doi: 10.1101/gr.234443.118 (PMC6442396; doi:10.1101/gr.234443.118)
Supplement: Supplemental Material [file supp_gr.234443.118_Supplemental_longranger-2.2.zip › longranger-2.2.tar.bz2!/lr2.2-public/lib/stan/cmdstan/stan/doc/api/html/agrad_2rev_2matrix_2sum_8hpp_source.html]

Stan: src/stan/agrad/rev/matrix/sum.hpp Source File


|  |  |
| --- | --- |
|  | Stan  2.5.0  probability, sampling & optimization |


- Main Page
- Related Pages
- Namespaces
- Classes
- Files

- File List
- File Members

All Classes Namespaces Files Functions Variables Typedefs Enumerations Enumerator Friends Macros Pages

- src
- stan
- agrad
- rev
- matrix

sum.hpp

Go to the documentation of this file.

1 #ifndef STAN\_\_AGRAD\_\_REV\_\_MATRIX\_\_SUM\_HPP

2 #define STAN\_\_AGRAD\_\_REV\_\_MATRIX\_\_SUM\_HPP

3

4 #include <vector>

5 #include <stan/math/matrix/Eigen.hpp>

6 #include <stan/math/matrix/typedefs.hpp>

7 #include <stan/agrad/rev/var.hpp>

8 #include <stan/agrad/rev/vari.hpp>

9 #include <stan/agrad/rev/matrix/typedefs.hpp>

10

11 namespace stan {

12  namespace agrad {

13

14  namespace {

15  class sum\_v\_vari : public vari{

16  protected:

17  vari\*\* v\_;

18  size\_t length\_;

19  inline static double var\_sum(const var \*v, size\_t length) {

20  double result = 0;

21  for (size\_t i = 0; i < length; i++)

22  result += v[i].vi\_->val\_;

23  return result;

24  }

25  template<typename Derived>

26  inline static double var\_sum(const Eigen::DenseBase<Derived> &v) {

27  double result = 0;

28  for (int i = 0; i < v.size(); i++)

29  result += v(i).vi\_->val\_;

30  return result;

31  }

32  public:

33  template<typename Derived>

34  sum\_v\_vari(const Eigen::DenseBase<Derived> &v) :

35  vari(var\_sum(v)), length\_(v.size()) {

36  v\_ = (vari\*\*)memalloc\_.alloc(length\_\*sizeof(vari\*));

37  for (size\_t i = 0; i < length\_; i++)

38  v\_[i] = v(i).vi\_;

39  }

40  template<int R1,int C1>

41  sum\_v\_vari(const Eigen::Matrix<var,R1,C1> &v1) :

42  vari(var\_sum(v1)), length\_(v1.size()) {

43  v\_ = (vari\*\*)memalloc\_.alloc(length\_\*sizeof(vari\*));

44  for (size\_t i = 0; i < length\_; i++)

45  v\_[i] = v1(i).vi\_;

46  }

47  sum\_v\_vari(const var \*v, size\_t len) :

48  vari(var\_sum(v,len)), length\_(len) {

49  v\_ = (vari\*\*)memalloc\_.alloc(length\_\*sizeof(vari\*));

50  for (size\_t i = 0; i < length\_; i++)

51  v\_[i] = v[i].vi\_;

52  }

53  virtual void chain() {

54  for (size\_t i = 0; i < length\_; i++) {

55  v\_[i]->adj\_ += adj\_;

56  }

57  }

58  };

59  }

60

67  template <int R, int C>

68  inline var sum(const Eigen::Matrix<var,R,C>& m) {

69  if (m.size() == 0)

70  return 0.0;

71  return var(new sum\_v\_vari(m));

72  }

73

74  }

75 }

76 #endif

vari.hpp

typedefs.hpp

typedefs.hpp

stan::length

size\_t length(const T &)

**Definition:** traits.hpp:159

stan::agrad::memalloc\_

memory::stack\_alloc memalloc\_

**Definition:** var\_stack.cpp:16

length\_

size\_t length\_

**Definition:** sum.hpp:18

stan::agrad::sum

fvar< T > sum(const Eigen::Matrix< fvar< T >, R, C > &m)

**Definition:** sum.hpp:14

v\_

vari \*\* v\_

**Definition:** sum.hpp:17

var.hpp

Eigen.hpp

stan::memory::stack\_alloc::alloc

void \* alloc(size\_t len)

Return a newly allocated block of memory of the appropriate size managed by the stack allocator...

**Definition:** stack\_alloc.hpp:168

stan::agrad::var

Independent (input) and dependent (output) variables for gradients.

**Definition:** var.hpp:27

stan::math::size

int size(const std::vector< T > &x)

**Definition:** size.hpp:11


---

[ Stan Home Page ]

*© 2011–2014,
Stan Development Team.*
